# Cross-species recognition of two porcine coronaviruses to their cellular receptor aminopeptidase N of dogs and seven other species

**DOI:** 10.1371/journal.ppat.1012836

**Published:** 2025-01-07

**Authors:** Yuyang Tian, Junqing Sun, Xiaohan Hou, Zhimin Liu, Zeao Chen, Xiaoqian Pan, Ying Wang, Jianle Ren, Ding Zhang, Bo Yang, Longlong Si, Yuhai Bi, Kefang Liu, Guijun Shang, Wen-xia Tian, Qihui Wang, George Fu Gao, Sheng Niu

**Affiliations:** 1 College of Veterinary Medicine, Shanxi Agricultural University, Jinzhong, China; 2 CAS Key Laboratory of Pathogen Microbiology and Immunology, Institute of Microbiology, Chinese Academy of Sciences (CAS), Beijing, China; 3 CAS Key Laboratory of Quantitative Engineering Biology, Shenzhen Institute of Synthetic Biology, Shenzhen Institute of Advanced Technology, Chinese Academy of Sciences (CAS), Shenzhen, China; 4 Shanxi Provincial Key Laboratory of Protein Structure Determination, Shanxi Academy of Advanced Research and Innovation, Taiyuan, China; University of New Mexico School of Medicine, UNITED STATES OF AMERICA

## Abstract

Porcine deltacoronavirus (PDCoV) and transmissible gastroenteritis coronavirus (TGEV), the two causative agents of porcine diarrhea, have been reported to be at risk of cross-species transmission, including to humans. However, the potential host range in which these two CoVs interact remains unclear. We screened 16 animal counterparts for porcine aminopeptidase N (APN), the receptor of PDCoV and TGEV, and found that APNs from eight of 17 animals could bind to the receptor-binding domains (RBDs) of PDCoV and TGEV. Furthermore, the animal APNs that could bind to the RBDs could mediate cellular infection by both viruses. Dog APN (dAPN) has been identified as the animal receptor with the highest capability to mediate the virus infection. We further resolved the complex structures of dAPN bound to the PDCoV RBD/TGEV RBD, respectively, establishing its divergent receptor-binding modes. We identified R325 of dAPN as an important residue in the PDCoV RBD-dAPN interaction, and found the central role of Q746 and T749 in dAPN in the interaction with the TGEV RBD. These findings provide the molecular basis of the potential cross-species transmission of these two porcine CoVs and shed light on future surveillance of these CoVs.

## Author summary

TGEV and PDCoV are common enteropathogens of swine with worldwide distribution, and they exhibited a capacity for cross-species transmission, including to humans. Here, we screened the binding abilities of these CoVs to the APN receptors from 17 species and the ability of different APNs to mediate viral infections to evaluate their potential cross-species transmission. The complex structures, TGEV RBD-dAPN and PDCoV RBD-dAPN, revealed distinct modes of interaction between these CoVs and their cellular receptor APN. Mutational analysis revealed that the R325, Q746 and N747-linked glycans on dAPN play critical roles in the interaction with PDCoV RBD and TGEV RBD, respectively. These findings provide the molecular basis for a better understanding of the potential cross-species transmission of these two porcine CoVs.

## Introduction

The zoonotic transmission of viruses from nonhuman animals poses significant threats to global public health and economic development [1, 2]. Coronaviruses (CoVs), categorized into alpha, beta, gamma, and delta genera (α-CoV, β-CoV, γ-CoV, δ-CoV) [3], cause mild to severe respiratory and gastrointestinal diseases in humans and animals, which has resulted in increasing attention and research priority [4–6]. Three members of β-CoVs, Middle East respiratory syndrome CoV (MERS-CoV), severe acute respiratory syndrome CoV (SARS-CoV), and SARS-CoV-2, are highly pathogenic and have all caused either epidemic or pandemics in the last two decades [7]. To date, six CoVs have been identified as infecting pigs, including four alpha-CoVs [(transmissible gastroenteritis virus (TGEV), porcine respiratory coronavirus (PRCoV), porcine epidemic diarrhea virus (PEDV), and swine acute diarrhea syndrome-coronavirus (SADS-CoV)], one beta-CoV [porcine hemagglutinating encephalomyelitis virus (PHEV)], and the porcine deltacoronavirus (PDCoV) [8]. Among them, TGEV and PRCV have been circulating in pigs for decades, whereas PDCoV is considered an emerging coronavirus. TGEV was initially reported in the USA in 1946 [9]. Since then, it has been identified in swine-producing areas worldwide, with continuous reports in China [10–12]. PRCoV, a variant of TGEV with a deletion of 621–681nt in the S protein, exhibits a shift in major tissue tropism from enteric to respiratory compared to the parental TGEV [13]. PDCoV, initially discovered in Hong Kong in 2012, emerged among swine herds with diarrhea in Ohio, USA, in 2014 [14, 15]. Recently, the detection of Hu-PDCoV and Canine CoVs (CCoVs) in humans has highlighted the risk of cross-species transmission of these zoonotic CoVs [5, 6, 16, 17].

Coronaviruses often break the host barrier and spread among other animals, sometimes in large epidemics or even pandemics [2, 4, 18, 19]. PDCoV can contain a wide range of hosts. In addition to being found to infecting pigs and humans, PDCoV can also experimentally infect chickens, calves, and mice [20–22]. Furthermore, dogs, cats, and foxes have been confirmed to be infected with TGEV [23–25]. However, whether other animals are at risk of infection with these porcine CoVs remains unknown, resulting in the need for early warning of the potential spillover of these CoVs.

Receptor binding is a prerequisite for viral transmission and infection [2, 3]. Multiple studies have shown the potential broad host range of SARS-CoV-2-related CoVs through assessing the binding of their receptor-binding domains (RBDs) to angiotensin-converting enzyme 2 (ACE2) orthologs from various species [26–30]. Aminopeptidase N (APN), a common receptor for HCoV-229E and TGEV, has been identified as an entry receptor for PDCoV [31–33]. Studies have provided evidence that PDCoV exhibits broad receptor utilization and can invade cells by utilizing APN from pigs, humans to chicken [33]. Therefore, evaluating the binding of the spike (S) RBDs of PDCoV and TGEV to APNs from various species could provide valuable clues for understanding the cross-species transmission of PDCoV and TGEV.

Here, we evaluated the interaction between two porcine CoV RBD proteins (PDCoV and TGEV) and 17APNs in different species. To further study the binding mechanism of these CoVs with dog APN (dAPN), we solved the cryo-electron microscopy (cryo-EM) structures of these CoV RBDs complexed with dAPN at the resolution of 3.05 Å and 2.86 Å, respectively. Our data revealed different binding modes between dAPN and the RBD of these viruses. These functional and structural results suggest broad receptor usage for both PDCoV and TGEV, calling for further surveillance to monitor porcine CoVs carried by other animals.

## Results

### The cross-species recognition of PDCoV to APN orthologs from 17 species

**Fig 1:**
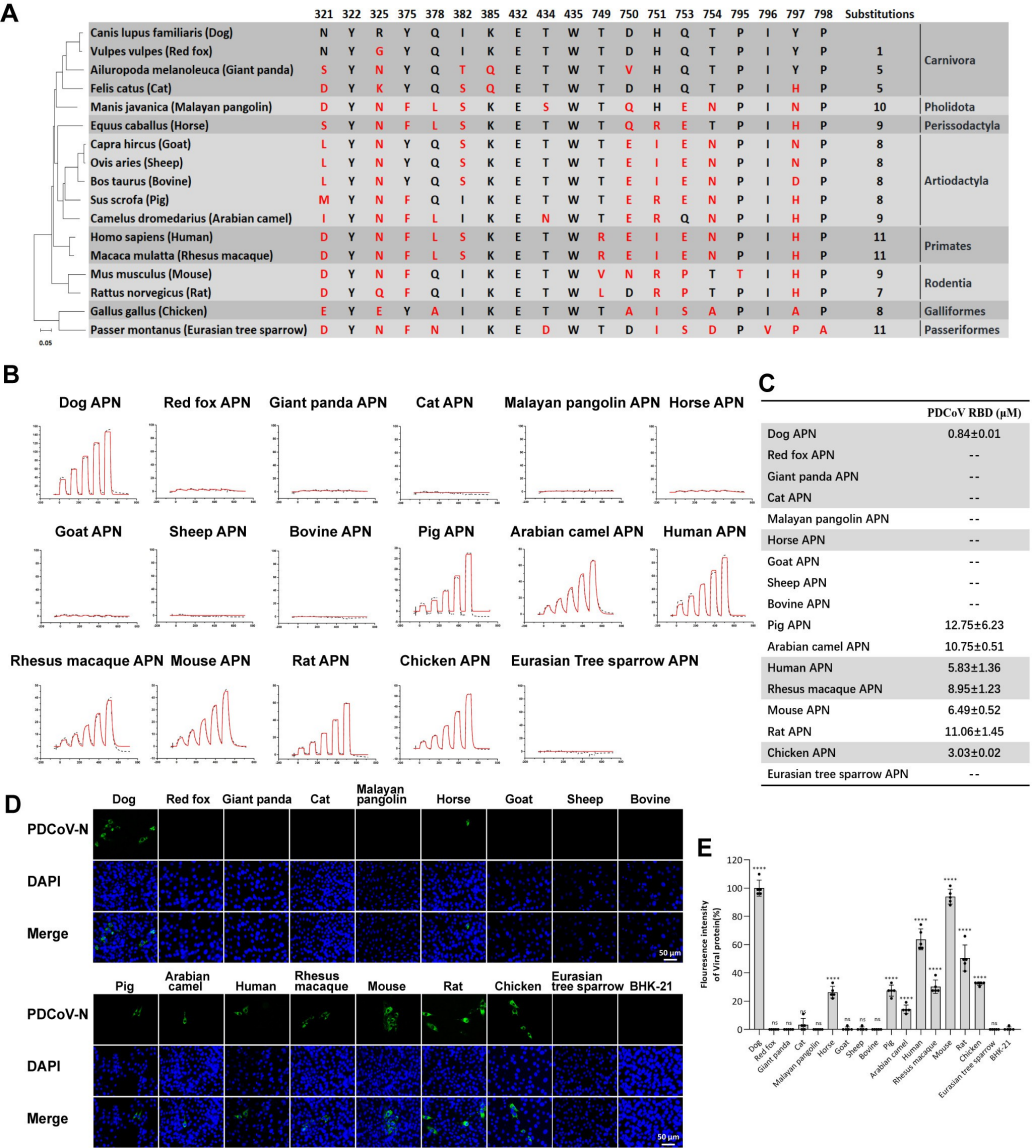
The cross-species recognition of PDCoV to APN orthologs from 17 species. (A) 19 residues of dAPN which are crucial in interacting with the PDCoV RBD are listed, respectively. Red letters indicate the substitutions in the APN of 16 animal species. The 17 species belonging to eight orders are shown in the right column. Phylogenetic tree based on APN amino acid sequences was generated using MEGA X. (B) The raw and fitted curves are displayed in dotted and solid lines, respectively. (C) The binding affinity between the PDCoV RBD and the 17 APNs is shown. Mean ± SD represents the mean and standard deviation of three independent experiments. (D) PDCoV infection in BHK-21 cells overexpressing the APNs from 17 species. Green fluorescence indicates BHK-21-APN cells infected with PDCoV. Wild-type BHK-21 cell is used as negative control. The scale bar indicates 50 μm. (E) The fluorescence intensity in (D) was determined by software Image J. Data are expressed as mean ± SD, n = 5. Error bars denote standard deviations for the samples. An unpaired two-tailed t-test was used to determine the statistical significance. *p < 0.05; ***p < 0.001; ****p < 0.0001; ns no significance.

To explore the potential for cross-species transmission of PDCoV and TGEV, we selected 16 animals besides humans, including domestic animals, companion pets, and some wild animals. Sequence alignment of the key residues in dAPN involved in the interaction with the PDCoV RBD from 17 species was performed. Based on the amino acid sequences of the APNs, we constructed a phylogenetic tree showing the genetic relationships of the 17 species (**Fig 1A**). These 17 species belong to eight orders, including Carnivora [*Felis catus* (Cat), *Canis lupus familiaris* (Dog), *Vulpes vulpes* (Red fox) and *Ailuropoda melanoleuca* (Giant panda)], Pholidota [*Manis javanica* (Malayan pangolin)], Perissodactyla [*Equus caballus* (Horse)], Artiodactyla [*Bos taurus* (Bovine), *Sus scrofa* (Pig), *Capra hircus* (Goat), *Ovis aries* (Sheep) and *Camelus dromedarius* (Arabian camel)], Primates [*Homo sapiens* (Human) and *Macaca mulatta* (Rhesus macaque)], Rodentia [*Mus musculus* (Mouse) and *Rattus norvegicus* (Rat)], Galliformes [*Gallus gallus* (Chicken)], Passeriformes [*Passer montanus* (Eurasian tree sparrow), which is the animal host of the CoV HKU17, the closest relative of PDCoV)] [14] (**Fig 1A**). The 19 key residues in dAPN responsible for binding to the PDCoV RBD were highlighted and compared with the 16 APN orthologs base on the complex structure in this study. The number of residue substitutions in APN orthologs ranged from 1 to 11, compared to dAPN. From the residue comparison of APNs, we found that the Y322, E432 and W435 sites were completely conserved among these 17 species (**Fig 1A**). Residues at positions 321, 325, 750, and 797 differed most frequently among the 17 APN orthologs.

We evaluated the binding characteristics between the 17 APN orthologs in these animals and PDCoV using a surface plasmon resonance (SPR) assay (**Fig 1B and 1C**). For PDCoV, the RBD protein can bind to APNs from eight species, including Primates (human and Rhesus macaque), Carnivora (dog), Rodentia (mouse and rat), Artiodactyla (pig and Arabian camel), and Galliformes (chicken). Specifically, the binding affinity between the APNs and PDCoV RBD was at the micromolar level. Among the APNs from different animals, the dAPN displayed the strongest binding affinity to PDCoV RBD, with the equilibrium dissociation constants (*K*_D_) calculated to be 0.84 ± 0.01 μM. Although pigs are the main host of PDCoV, the interaction of pig APN (pAPN) with PDCoV RBD in this study is relatively low, with the *K*_D_ calculated to be 12.75 ± 6.23 μM, which is similar to that of Arabian camel APN (acAPN). Rhesus macaque APN (rmAPN) interacts with PDCoV RBD at a strength similar to that of human APN (hAPN). In rodents, the binding affinity of mouse APN (mAPN) to the PDCoV RBD is ~2-fold stronger than that of rat APN (rAPN). Chicken is thought to be susceptible to PDCoV, and the binding affinity of chicken APN (chAPN) to the PDCoV RBD was calculated to be 3.03 ± 0.02 μM.

To determine whether PDCoV infect cells via binding to APNs from different species, PDCoV were used to infect BHK-21 cells with the expression of APNs from different species. Consistent with the results of the SPR assays, all these animal APNs (dAPN, pAPN, acAPN, hAPN, rmAPN, mAPN, rAPN and chAPN) that bind to PDCoV RBD can mediate the infection of PDCoV into BHK-21 cells (**Fig 1D and 1E**), but PDCoV was unable to infect the BHK-21 cells without the expression of APN. Among the APNs from different animals, dAPN also mediated the strongest infection efficiency of PDCoV, which is consistent with its strongest binding affinity. Although the horse APN (hsAPN) displays no detectable binding with the PDCoV RBD, it could still mediate the infection of PDCoV at a low level (**Fig 1D and 1E**).

### The cross-species recognition of TGEV to APN orthologs from 17 species

**Fig 2:**
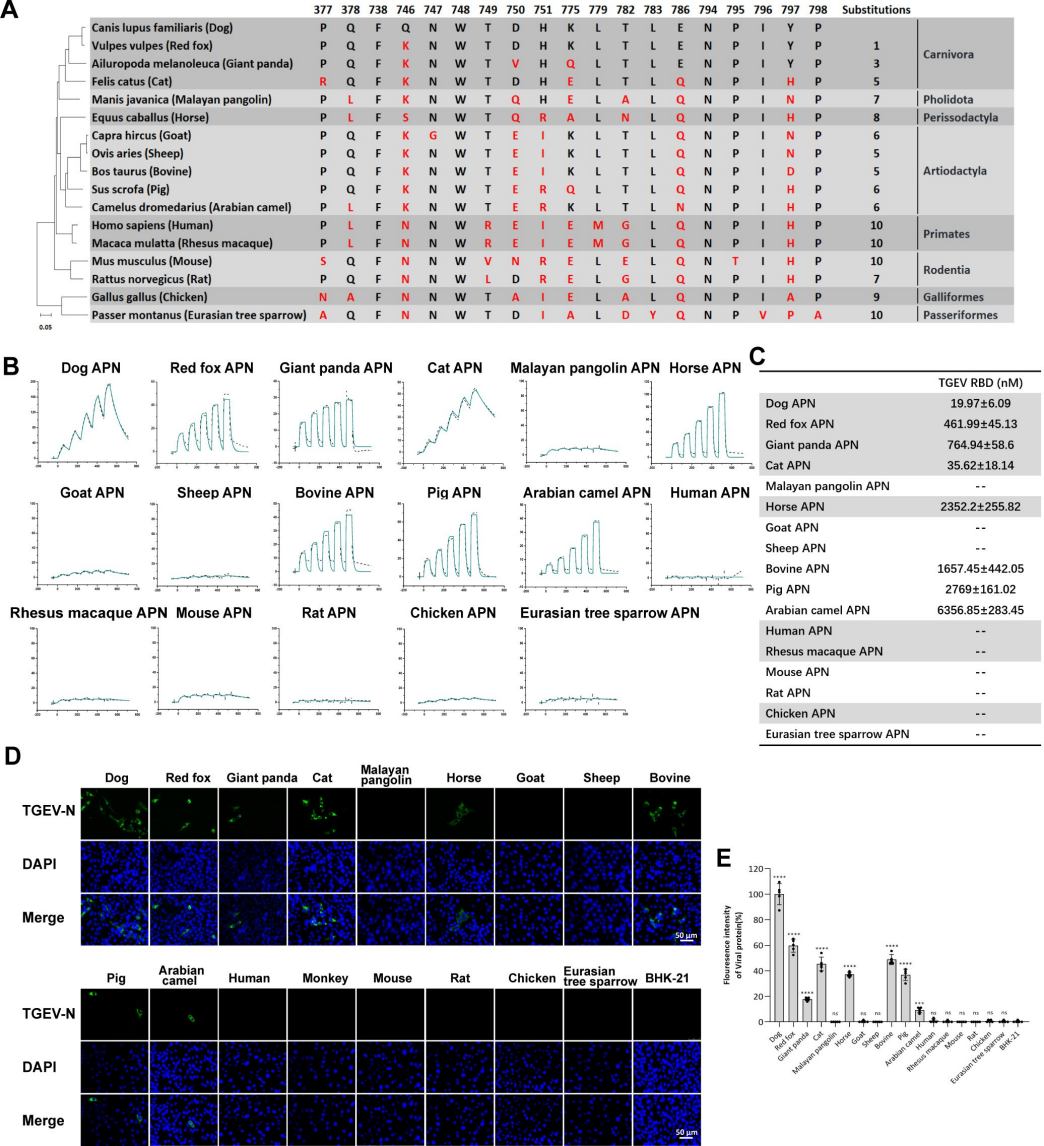
The cross-species recognition of TGEV to APN orthologs from 17 species. (A) 19 residues of dAPN which are crucial in interacting with the TGEV RBD are listed, respectively. Red letters indicate the substitutions in the APN of 16 animal species. The 17 species belonging to eight orders are shown in the right column. (B) The raw and fitted curves are displayed in dotted and solid lines, respectively. (C) The binding affinity between the TGEV RBD and the 17 APNs is shown. Mean ± SD represents the mean and standard deviation of three independent experiments. (D) TGEV infection in BHK-21 cells overexpressing the APNs from 17 species. Green fluorescence indicates BHK-21-APN cells infected with TGEV. Wild-type BHK-21 cell is used as negative control. The scale bar indicates 50 μm. (E) The fluorescence intensity in (D) was determined by software Image J. Data are expressed as mean ± SD, n = 5. Error bars denote standard deviations for the samples. An unpaired two-tailed t-test was used to determine the statistical significance. *p < 0.05; ***p < 0.001; ****p < 0.0001; ns no significance.

Sequence alignment of the key residues in dAPN involved in the interaction with the TGEV RBD from 17 species was performed (**Fig 2A**). The 19 residues in dAPN responsible for binding to the TGEV RBD were highlighted and compared with the 16 APN orthologs base on the complex structures in this study. The number of residue substitutions in APN orthologs ranged from 1 to 10, compared to dAPN. From the residue comparison of APNs, we found that the F738, W748, and N794 sites were completely conserved among these 17 species (**Fig 2A**). Residues at positions 746, 750, and 797 differed most frequently among the 17 APN orthologs.

We also evaluated the binding characteristics between the 17 APN orthologs and TGEV RBD using the SPR assay (**Fig 2B and 2C**). Overall, the PDCoV and TGEV RBD displayed different receptor-binding spectra to APNs from different animals. For TGEV, the RBD could bind to APNs from eight species, including Carnivora (dog, cat, red fox, and giant panda), Artiodactyla (pig, bovine, and Arabian camel), and Perissodactyla (horse). Specifically, pAPN, the receptor of the main host of TGEV, displayed apparent binding affinity to TGEV RBD with the *K*_D_ value of 2.77 ± 0.16 μM. The binding affinity between the TGEV RBD and bovine (bAPN) and hsAPN is similar to that of pAPN. In contrast, the binding affinity of acAPN to the TGEV RBD was ~3-fold weaker. Interestingly, dAPN, which has the highest affinity to PDCoV RBD, also has a strong affinity to TGEV RBD, with the *K*_D_ value calculated to be 19.97 ± 6.09 nM. In addition, APNs from other animals belonging to Carnivora interacted with the TGEV RBD with an approximately 4- to 140-fold stronger binding affinity than that of pAPN. Bovine and sheep APNs differ by a single residue at position 797 (N for sheep, D for bovine) among the residues interacting with TGEV RBD, yet they exhibit significantly different binding to TGEV. Therefore, we introduced two mutations (bovine APN-D797N and sheep APN-N797D) to explore the role of residue 797 in TGEV binding. Mutational analysis showed that wild-type sheep APN have no detectable interaction with TGEV RBD, but the N797D mutant acquired the high-binding affinity with TGEV RBD (**S1 Fig**). In addition, bovine APN-D797N displayed 5-fold weaker binding affinity with TGEV RBD than wild-type bovine APN (**S1 Fig**).

To determine whether TGEV infect cells via binding to APNs from different species, TGEV were used to infect BHK-21 cells with the expression of APNs from different species. Consistent with the results of the SPR assays, all these animal APNs (dAPN, red fox APN, giant panda APN, cat APN, bAPN, hsAPN, pAPN and acAPN) that bind to TGEV RBD can mediate the infection of TGEV into BHK-21-APN cells (**Fig 2D and 2E**), but TGEV was unable to infect the BHK-21 cells without the expression of APN. Notably, dAPN also mediated the strongest infection efficiency of TGEV (**Fig 2D and 2E**).

### The overall structures of dAPN complexed with PDCoV RBD or TGEV RBD

**Fig 3:**
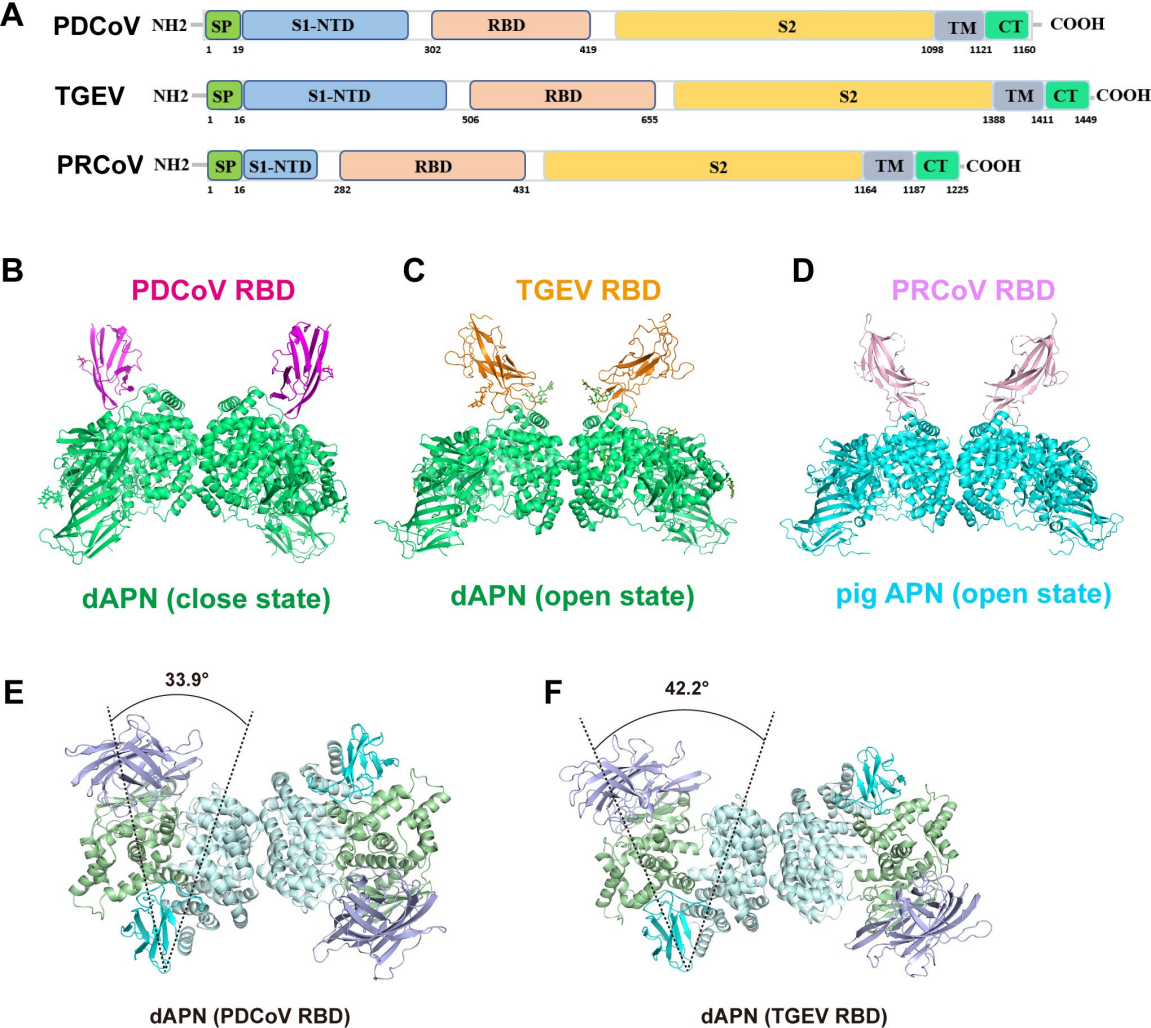
Overall structure of dAPN in complex with PDCoV RBD or TGEV RBD. (A) Schematic diagram of the S protein ectodomain of PDCoV, TGEV and PRCoV. S1-NTD, N-terminal domain of S1. RBD, receptor binding domain. (B) The complex structure of PDCoV RBD bound to dAPN. PDCoV RBD and dAPN are colored in magenta and green, respectively. (C) The complex structure of TGEV RBD bound to dAPN. TGEV RBD and dAPN are colored in orange and green, respectively. (D) The complex structure of PRCoV RBD bound to pAPN (PDB: 4F5C) [35]. PRCoV RBD and pAPN are colored in pink and cyan, respectively. (E and F) Ribbon representation of the closed (dAPN-PDCoV RBD) and open (dAPN-TGEV RBD) dimeric dAPN structures. The APN domains colored in lightblue (Domain I), palegreen (Domain II), cyan (Domain III) and palecyan (Domain IV). The angle indicates the swing movement of the Domain I-II-III module toward Domain IV after ectodomain closure in each monomer of the APN dimer, with Domain IV fixed by dimerization.

All RBDs of the three porcine CoVs (PDCoV, TGEV, and PRCoV) are located at the C terminus of the S1 subunit of the S protein (**Fig 3A**). Previous research has demonstrated that APNs from pigs, humans, and chickens can be used as receptors by PDCoV to infect cells [34]; however, only the structural basis of the PDCoV RBD bound to pAPN and hAPN has been resolved [34]. In addition to infecting pigs, TGEV has also been reported to infect dogs [23]. In this study, dAPN was identified as a receptor with a strong binding capacity to the PDCoV and TGEV RBD. To further investigate the molecular basis of dAPN interaction with PDCoV and TGEV RBD, the complex structures of PDCoV RBD-dAPN and TGEV RBD-dAPN were determined with a resolution of 3.05 Å and 2.86 Å, respectively, using cryo-EM (**S1 Table, S2 and S3 Figs**).

Similar to the published PDCoV RBD-hAPN structure [34], the complex structures of dAPN with PDCoV RBD showed that two PDCoV RBD molecules bind to the dimeric dAPN with its receptor-binding motifs (RBMs) composed of 4 β-strands and 2 connecting loops (**Fig 3B**). The structure of the PDCoV RBD-pAPN complex contains only one PDCoV RBD molecule binding to dimeric pAPN (**S4C Fig**), which may be related to the weak binding ability of pAPN to the RBD. Superimposition of the structure of the PDCoV RBD-dAPN onto that of PDCoV RBD-hAPN and PDCoV RBD-pAPN yields the root mean square deviation (RMSD) of 1.22 Å (866 Cα atoms) and 1.11 Å (877 Cα atoms), respectively, indicating the three APNs have highly conserved structures and similar mode in the interaction with the PDCoV RBD (**S4D and S4E Fig**). In the TGEV RBD-dAPN complex, the TGEV RBD, like the PRCoV RBD, adopts a β-barrel structure with two ligand binding loops. The TGEV RBD-dAPN complex displays increased divergence with PRCoV RBD-pAPN [35] (RMSD = 1.50 Å for 841 Cα atoms) (**S4F Fig**).

### Different binding modes of dAPN to PDCoV RBD and TGEV RBD

**Fig 4:**
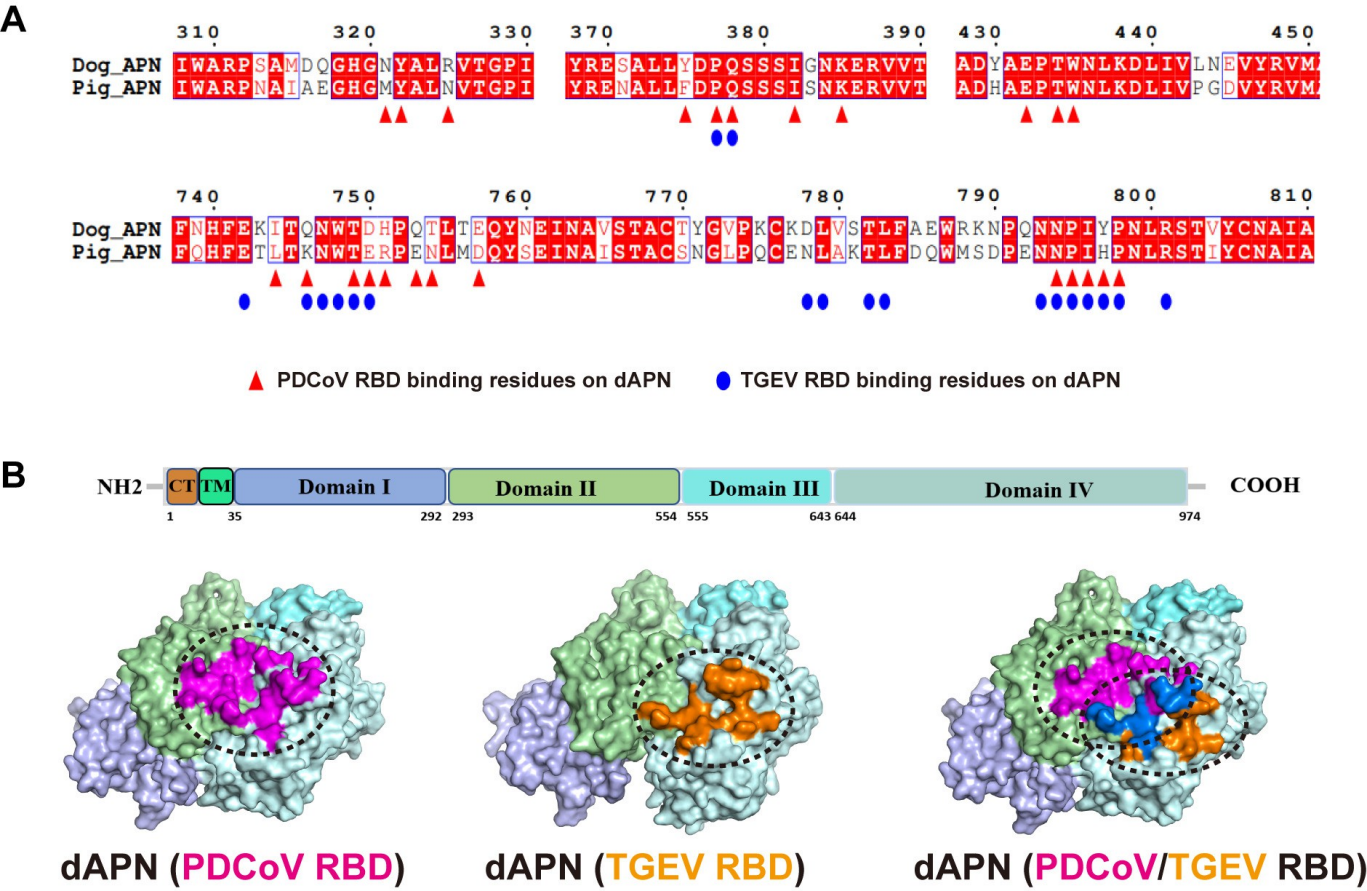
Comparison of PDCoV RBD and TGEV RBD binding sites. (A) Sequence alignment of the RBD sequences from the PDCoV and TGEV. The red triangles indicate the binding sites of the PDCoV RBD to dAPN, and the blue ellipses indicate the binding sites of the TGEV RBDs to dAPN. (B) The structure of dAPN displayed in surface view. Residues that interact with the PDCoV RBD and TGEV RBD are marked, respectively.

The dAPN, like hAPN and pAPN, can be divided into four subdomains, among which Domains II and IV interact with the RBDs of PDCoV and TGEV (**Fig 4**). However, the binding sites of the PDCoV RBD on dAPN are significantly different from those of the TGEV RBD (**Fig 4A**), indicating different receptor-binding modes. Here, the open conformation of dAPN is identified in the TGEV RBD-dAPN complex structure, in which Domain IV is distant from Domain II; thus, the catalytic site could be exposed (**Fig 3C and 3F**). Conversely, a closed conformation of dAPN is observed in the PDCoV RBD-dAPN complex, in which the four domains of dAPN are shaped like a clasped hand, restricting substrate access to the enzyme active site (**Fig 3B and 3E**). The binding sites of the PDCoV RBD are located in Domains II and IV of dAPN, and these two domains contribute 40% and 60% of the total buried surface area (BSA) of dAPN, respectively (**Fig 4B**). However, the binding sites of the TGEV RBD are mainly located in Domain IV, and Domain II contributed little (9%) to the BSA of the TGEV RBD (**Fig 4B**).

**Fig 5:**
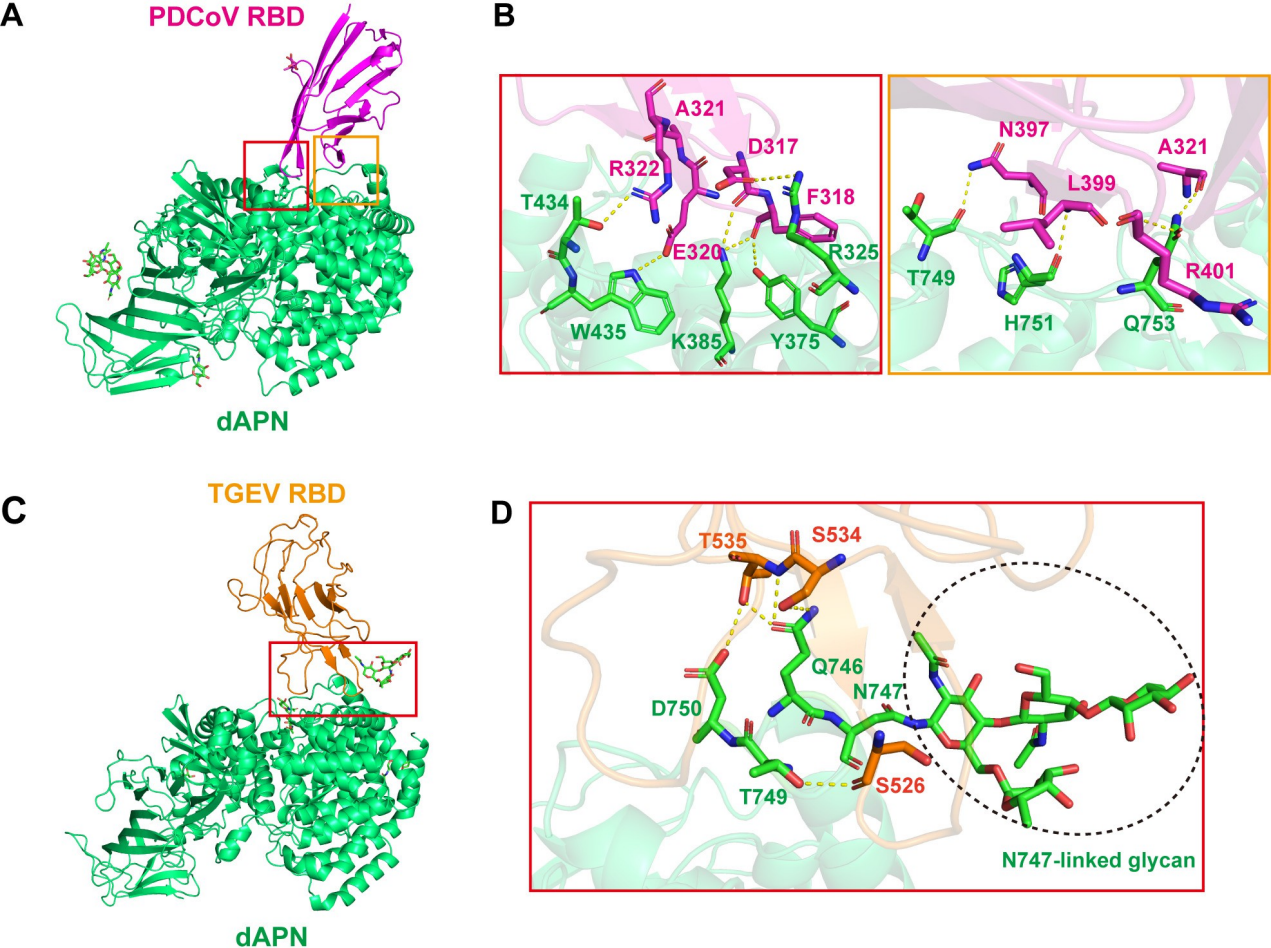
The complex structures of PDCoV RBD and TGEV RBD bound to dAPN. (A) The overall complex structures of dAPN bound to the PDCoV RBD. The binding between the PDCoV RBD and dAPN is mainly composed of two patches of interactions, and patch 1 and patch 2 are indicated in red and orange boxes, respectively. (B) Detailed interaction of dAPN with the PDCoV RBD in patch 1 and patch 2. Residues involved in the H-bonds are shown as yellow dotted lines with a cutoff of 3.5 Å. (C) The overall complex structures of dAPN bound to the TGEV RBD. (D) Detailed interaction of dAPN with the TGEV RBD. Residues involved in the H-bonds are shown as yellow dotted lines with a cutoff of 3.5 Å.

Key residues contributing to hydrogen bonds and van der Waals (vdw) interactions between dAPN and the RBDs of PDCoV and TGEV are also identified and labeled (**S2 and S3 Tables**, **Fig 5**). Overall, the total number of interactions of dAPN with the RBDs of PDCoV and TGEV are 107 and 224, including 10 and 5 hydrogen bonds, respectively (**S2 and S3 Tables**). A total of 19 residues of dAPN contact the PDCoV RBD, and the residues (R325, Y375, K385, T434, W435, T749, H751, and Q753) of dAPN form a hydrogen bond network with amino acids (D317, F318, R322, E320, N397, L399, A321, and R401) from the extending loops of the PDCoV RBD. For the TGEV RBD-dAPN complex structure, 19 residues of dAPN contacted TGEV RBD, and the residues (Q746, T749, and D750) of dAPN form a hydrogen bond network with amino acids (S526, S534, and T535) from TGEV RBD.

To explore the biological significance of the open/close conformation of APN when binding TGEV/ PDCoV RBDs, we introduced a mutant of pAPN with two cysteine substitutions at positions 464 and 929, which can form a disulfide bond, thereby stabilizing the APN in a closed conformation and inactivating its enzymatic activity [36]. The SPR assay showed that the binding affinity of TGEV RBD to the S464C-S929C mutant was ~6-fold weaker than that of wild-type pAPN, whereas the binding affinity of PDCoV to the S464C-S929C mutant was 4-fold stronger than that of wild-type pAPN (**S5A Fig**). The virus infection assays also indicated that the pAPN-S424C-S929C mutant significantly reduced the infection efficiency of TGEV than that of wild-type pAPN (**S5B Fig**), while the pAPN-S424C-S929C mutant significantly increased the infection efficiency of PDCoV than that of wild-type pAPN (**S5C Fig**).

### Identification of key residues responsible for the stronger binding of dAPN to PDCoV RBD

**Fig 6:**
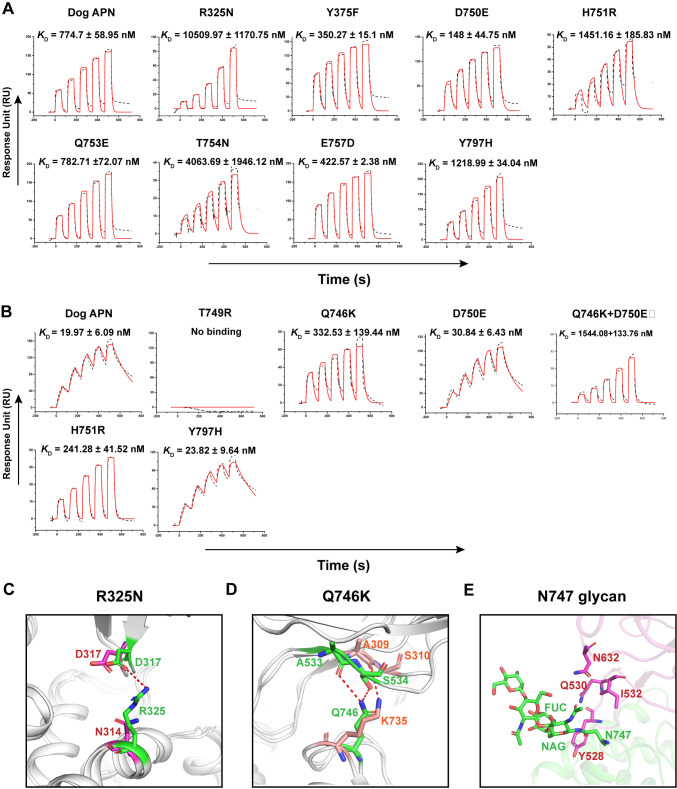
Binding affinity assay between dAPN mutants and the RBDs of PDCoV and TGEV by SPR. (A and B) The hFc-tagged dAPN mutants were captured by protein A chip, and sequentially tested the binding with serially diluted PDCoV RBD (A) or TGEV RBD (B). The binding affinities of the APN mutants to the TGEV RBD and PDCoV RBD are shown. Mean ± SD represents the mean and standard deviation of three independent experiments. (C) The structural details of PDCoV RBD-dAPN (green) and PDCoV RBD/pAPN (magenta) around the sites referred above each picture. (D) The structural details of TGEV RBD-dAPN (green) and PRCoV RBD/pAPN (pink) around the sites referred above each picture. Residues involved in the interaction are represented as sticks, and H-bonds are represented by red dashes. (E) The interaction of N747-linked glycan in dAPN (green) with TGEV RBD (magenta).

Next, we explored the mechanism that leads to stronger binding of PDCoV RBD to dAPN than to pAPN. Here, we focused on the APN substitution at the binding interface. Specifically, compared with the pAPN, the dAPN possesses eight substitutions on the PDCoV binding interface, namely N325R, F375Y, E750D, R751H, E753Q, N754T, D757E and H797Y. Therefore, we introduced eight mutations (R325N, Y375F, D750E, H751R, Q753E, T754N, E757D, and Y797H) to dAPN to mimic the key variant residues of pAPN binding to the PDCoV RBD (**Fig 6**). Mutational analyses were conducted by transiently expressing dAPN carrying different mutations in HEK293T cells.

For the PDCoV RBD, the binding affinities of the two mutants (dAPN-Q753E and dAPN-E757D) were similar to those of the wild-type dAPN. In comparison, the binding affinity of PDCoV RBD to the Y375F mutant was ~2-fold stronger. Moreover, the binding affinity of PDCoV RBD to the H751R and T754N mutants was ~2-fold and ~5-fold weaker, respectively, and that of the R325N mutant was ~14-fold weaker, which explains the stronger binding of dAPN to PDCoV RBD. We aligned the dAPN-PDCoV and pAPN-PDCoV complex structures and analyzed the reason for the great change in the affinity of the R325N mutant. The N314 residue in pAPN only forms vdw contacts with the PDCoV RBD. In contrast, the side chain of the R325 residue in dAPN is longer, allowing it to form a stronger interaction with the PDCoV RBD and form hydrogen bond and salt bridge and several vdw contacts with the D317 residue (**Fig 6C**).

### Identification of key residues responsible for the stronger binding of dAPN to the TGEV RBD

Then we explored the mechanism that leads to stronger binding of the TGEV RBD to dAPN than to pAPN. At the TGEV RBD-binding interface, dAPN possesses six substitutions compared to the pAPN: K746Q, E750D, R751H, K775Q, E786Q and H797Y. Therefore, we introduced five mutations (Q746K, D750E, Q746K-D750E, H751R and Y797H) of dAPN to mimic the key variant residues of pAPN binding to the TGEV RBD (**Fig 6**). The mutant T749R, which can lose the N747-linked glycan, was also included to explore the role of the N747-glycosylation in binding to TGEV RBD.

The mutant T749R, which can lose the N747-linked glycan, lost its high binding affinity with the TGEV RBD compared to wild-type dAPN (**Fig 6E**). Notably, all of the APN receptor holding no T749 from animals (human, Rhesus macaque, mouse and rat) could not interact with TGEV RBD. Therefore, interaction with the N747-linked glycan is crucial for the binding of dAPN to the TGEV RBD, which also plays a critical role in the host range of TGEV. The glycosylation sites at positions 747, 748, and 749, which form a key loop region for binding to the TGEV RBD. Whether the adjacent positions 746 and 750 have an overall effect on the binding affinity requires further evaluation. The SPR results showed that the binding affinities of the D750E and Y797H mutants are similar to that of wild-type dAPN. In comparison, the binding affinity of the TGEV RBD to the Q746K and Q746K-D750E mutants is approximately 16- and 77-fold weaker, respectively. Structural comparison of the two complexes, dAPN-TGEV RBD and pAPN-PRCoV RBD, was conducted to analyze the reasons for the significant difference in the affinity. The Q746 residue in dAPN could form hydrogen bonds with A533 and S534 in the TGEV RBD, whereas the K735 residue in pAPN could only form one hydrogen bond with A309 in the PRCoV RBD, resulting in a significant reduction in affinity following the Q746K mutation (**Fig 6D**).

## Discussion

The COVID-19 pandemic caused by SARS-CoV-2 makes us more concerned about the potential cross-species transmission of various CoVs. Two porcine CoVs, PDCoV and TGEV, have caused serious losses to the pig industry and pose a risk of cross-species transmission to multiple animals or might contribute some gene fragments for recombination [20, 21, 23, 37]. Notably, the newly discovered Hu-PDCoV can cause respiratory diseases in children [6]. Thus, prospective studies on the possible host ranges of these two porcine CoVs are required.

Receptor binding is a prerequisite for viral transmission and infection [2, 3, 38]. By assessing the binding capacity between the RBDs of different CoVs and receptors from different animals, the potential host range of multiple CoVs (eg: SARS-CoV-2, bat CoVs RaTG13 and RshSTT182, pangolin CoVs GX/P2V/2017 and GD/1/2019) has been well established [27, 29, 39]. Using a similar strategy, we constructed and expressed APN orthologs from 17 species and evaluated the interaction between the RBDs of PDCoV and TGEV and 17 APNs. PDCoV RBD was found to interact with APNs from eight species, including Primates (human and Rhesus macaque), Carnivora (dog), Rodentia (mouse and rat), Artiodactyla (pig and Arabian camel), and Galliformes (chicken). Meanwhile, virus infection assays showed that all these animal APNs binding to PDCoV RBD can mediate the infection of PDCoV into cells. Consistent with this, it has been reported that PDCoV could infect cells transiently expressing pig, human, and chicken APNs [22, 33, 40]. These results also evoke concerns about the potential cross-species transmission of PDCoV, and further spill-over to humans.

Furthermore, the TGEV RBD also interacted with APNs from eight species, including animals from Carnivora (dog, cat, red fox, and giant panda), Artiodactyla (pig, bovine, and Arabian camels), and Perissodactyla (horse), but not with human APN. The cell entry assays also showed that the eight animal APNs binding to TGEV RBD can mediate the infection of TGEV into cells, suggesting the possible cross-species transmission of TGEV. Continuous surveillance of TGEV in animals with APNs that can interact with RBDs is required.

Dogs are among the most popular domestic pets worldwide and have the most frequent contact with humans. Among the 17 APN orthologs, The RBDs of PDCoV and TGEV displayed the strongest binding affinities to dAPN. Although there are currently no reports of PDCoV infecting dogs, the high affinity of dAPN for the PDCoV RBD increases the possibility of PDCoV infection in dogs. The alphacoronavirus-1 species, TGEV, CCoV-II, and feline CoV (FCoV) II have reportedly originated from CCoV-I and FCoV-I through gene loss and recombination [41–43]. Meanwhile, TGEV has also been reported to infect dogs [23], which explain the high binding capacity of dAPN to the TGEV RBD. Recently, two novel canine-feline recombinant alphacoronavirus (CCoV-HuPn-2018 and HuCCoV_Z19Haiti) were isolated from a human patient [5, 17]. Given the close proximity of dogs to pigs in many farms, the possibility of recombination between CoV lineages in dogs poses a significant risk of interspecies transmission or the emergence of novel CoVs.

Previous studies have shown that PDCoV and PRCoV can bind to distinct sites on pAPN [34, 35]. The cryo-EM structures of dAPN complexed with PDCoV and TGEV RBD are reported here, indicating that the binding sites of PDCoV RBD on dAPN are significantly divergent from those of TGEV RBD. Previous studies have shown that free APN adopts open, intermediate, or closed conformations. Substrates can bind to the active site of APN through an open conformation and then undergoes hydrolysis to form a closed conformation [36]. In this study, an open conformation of dAPN was also observed in the TGEV RBD-dAPN complex structure; thus, the catalytic site was exposed. However, similar to hAPN and pAPN [34], the dAPN binds to PDCoV RBD using the close conformation, restricting peptide substrate access to the enzyme active site. Meanwhile, the SPR and virus infection assays also demonstrated that maintaining pAPN in the closed conformation could enhance the binding and infection of PDCoV, but weaken the binding and infection of TGEV (**S5 Fig**), which indicates the biological significance of different APN conformations in the viral infections.

The structures of the PDCoV RBD complexed with hAPN/pAPN were recently reported [34], and the vital role of multiple residues in APN in PDCoV infection was identified. In this study, structural analysis revealed that R325 of dAPN plays critical roles in the interaction networks with the PDCoV RBD. Sequence alignment of APNs from various animals revealed that the R325 region are highly polymorphic. Mutational analysis in this study suggested that dAPN receptor carrying the R325N variant have a greatly weakened binding capacity for the PDCoV RBD, which supports the important role of the residue R325 in the potent binding affinity of dAPN to the PDCoV RBD.

The glycosylation of APN plays a crucial role in the interactions between receptor and PRCoV and CCoV-HuPn-2018 [35, 44]. Similarly, the SPR assay supported that the removal of glycosylation at N747, dAPN-T749R, lost its interaction with TGEV RBD. The glycosylation sites at positions 747, 748, and 749, which form a key loop region for binding to the TGEV RBD. For the complex structure of TGEV RBD-dAPN, structural analysis revealed that the residue Q746 of dAPN contributes significantly to the formation of hydrogen bonds (**Fig 6D**). The Q746K mutant, which exists in many species, attenuates the binding capacity of dAPN, indicating the central roles of Q746 in the interaction network. Although the D750E mutation has a similar affinity to the wild-type dAPN, Q746K-D750E reduces the affinity for TGEV RBD by ~5-fold compared to the Q746K mutation. This suggests that the double mutations at the adjacent positions may alter the loop structure located at the glycosylation site, thereby reducing binding to TGEV RBD. This also raises the possibility that extensively diversified APN have profound implications for the evolutionary adaptation of the two porcine CoVs to other potential host receptors.

In summary, we identified broad cross-species recognition by TGEV and PDCoV using APN orthologs. The structures of the PDCoV RBD/TGEV RBD complexed with dAPN with the strongest binding affinity were determined, revealing divergent receptor-binding modes of these two porcine CoVs. The R325 and Q746, D750 N747-linked glycans on dAPN play critical roles in the interaction with PDCoV RBD and TGEV RBD, respectively. These findings provide directions for the potential cross-species transmission of these two porcine CoVs and shed light on future surveillance of these CoVs.

## Materials and methods

### Cells

HEK293F cells were cultured in SMM 293-TII medium (Sinobiological) at 37°C and 5% CO_2_, shaking with 140 rpm. HEK293T and BHK-21 cells were cultured in Dulbecco’s Modified Eagle medium (DMEM) containing 10% fetal bovine serum (FBS) at 37°C and 5% CO_2_. PDCoV CHN/SX-Y/2023 strain (GenBank accession No. PQ373831.2) and TGEV SX413 strain (GenBank accession No. PQ603017) were isolated from the piglets suffering severe diarrhea and passaged in LLC-PK1 cells with DMEM supplemented with 10 μg/ml trypsin.

### Gene cloning

The coding sequences of full-length APN orthologs from 17 animals (accession numbers are shown in **S4 Table**) were synthesized and cloned into pEGFP-C1, and the ectodomains of all APNs fused with the Fc domain of human IgG (hFc) were synthesized and cloned into pCAGGS vectors, respectively. The coding sequences of PDCoV RBD (residues 297–428, GenBank accession no. MW685624.1), TGEV RBD (residues 496–670, GenBank accession no. AJ271965) and dAPN ectodomain were cloned into pCAGGS vectors with a C-terminal six histidine tag. An interleukin-2 signal peptide sequence was added to the N-terminus of each construct to facilitate protein secretion.

### Protein expression and purification

The PDCoV RBD and TGEV RBD with his tag were expressed and purified from the culture supernatants of HEK293F cells using a His-Trap Excel column (GE Healthcare) and further purified by Superdex 200 Increase 10/300 GL column (GE Healthcare). Purified proteins were stored in protein buffer (20 mM Tris-HCl, pH 8.0 and 150 mM NaCl). To prepare the hFc-tagged APN and the APN mutant proteins, the pCAGGS plasmids containing the coding sequences of APNs were transiently transfected into HEK293T cells. 48 h later, supernatant containing the indicated protein were collected, concentrated and then used for SPR assays.

### SPR assay

The supernatants containing 17 hFc-tagged APNs or the APN mutants were respectively captured on flow cell 2 of the protein A sensor chip (GE Healthcare) at more than 1000 response units. Flow cell 1 was used as the negative control. Then, serially diluted PDCoV RBD and TGEV RBD proteins flowed over the chip in PBST buffer. Response Units (RU) were measured with a BIAcore 8K (GE Healthcare) in single-cycle mode. The antibodies were regenerated with 10 mM glycine-HCl (pH 1.7). The equilibrium dissociation constants (*K*_D_) of each pair of interactions were calculated using BIAcore 8K Evaluation Software (GE Healthcare) by fitting to a 1:1 Langmuir binding model. Data is presented as mean ± SD of three independent results.

### Generation of stable BHK-21 cell lines expressing APN from different species

APN wild-type or the mutant genes was individually cloned into pCDNA 4.0 plasmid with a C-terminal Flag tag. The plasmids were used to transfect BHK-21 cells using polyethyleneimine (Yeasen) according to the manufacturer’s instructions. After incubation for 24 h, cells were treated with 200 μg/ml Zeocin (Invitrogen) for 1 week. After that, single colony cells were collected.

### Immunofluorescence assay

For IFA, cells infected with PDCoV/TGEV in 24-well plates were washed thrice with PBS, then fixed with 4% paraformaldehyde for 30 min at room temperature. After washing thrice with PBS, the cells were permeabilized with Triton X-100 (0.1%) for 10 min, followed by washing thrice with PBS and blocked with 5% skimmed milk for 1 h at 37°C. For PDCoV, cells were rinsed with PBS three times, then incubated with anti-PDCoV N monoclonal antibody at 1:1000 dilution for 1 h at 37°C, followed by incubation with FITC conjugated goat Anti-mouse IgG (Abcam, Cat: ab6911) at 1:1000 dilution for 1 h at 37°C. For TGEV, cells were rinsed with PBS three times, then incubated with anti-TGEV N polyclonal antibody at 1:400 dilution for 1 h at 37°C, followed by incubation with FITC conjugated goat Anti-mouse IgG (Abcam, Cat: ab6911) at 1:1000 dilution for 1 h at 37°C. Finally, cells were stained with 0.01% 4′,6-diamidino-2-phenylindole (DAPI), and washed three times. Fluorescent images were collected with a fluorescence microscope (Leica). The fluorescence intensity was determined by software Image J. Each group contains 5 replicates. Statistical significance was determined by a two-sided unpaired Student’s t-test.

### Cryo-EM sample preparation and data acquisition

To prepare the cryo sample, the PDCoV RBD-dAPN complex and the TEGV RBD-dAPN complex samples were both vitrified using a Vitrobot Mark IV (ThermoFisher Scientific) plunge freezing device. The PDCoV RBD-dAPN complex sample (4.0 μL, 1.5 mg/mL) was applied to an Au Quantifoil 1.2/1.3 holey carbon grid with glow discharged for 40 seconds. The TEGV RBD-dAPN complex sample (4.0 μL, 1.8 mg/mL) was applied to a 40 seconds glow discharged Au Quantifoil 1.2/1.3 holey carbon grid. Grids above were then blotted using different conditions (blot time 6 s and blot force -10 for PDCoV RBD-dAPN complex; blot time 6 s and blot force -4 for the TEGV RBD-dAPN complex) at a temperature of 4°C and a humidity level of >99% and plunge frozen into liquid ethane.

The prepared grids were transferred to a 300 kV Titan Krios transmission electron microscope equipped with Gatan K3 detector and GIF Quantum energy filter. Movies were collected at 105,000× magnification with a calibrated pixel size of 0.69 Å over a defocus range of -1.0 μm to -2.0 μm in super resolution counting mode with a total dose of 60 e^-^/Å^2^ using EPU (ThermoFisher Scientific) automated acquisition software.

### Image processing

The detailed data processing workflow is summarized in **S2 and S3 Figs**. All the raw dose-fractionated images stacks were 2× binned, aligned, dose-weighted and summed using MotionCor2 [45]. The contrast transfer function (CTF) estimation, particle picking and extraction, 2D classification, ab initio model generation, 3D refinements were performed in cryoSPARC v.4.5.1 [46].

For the PDCoV RBD-dAPN complex, a total of 21,412 micrographs were collected for this dataset, from which 4,127,192 initial particles were picked and extracted with the box size of 400 pixels. After five rounds of iterative 2D classification, a clean set of 1,086,603 particles were used to generate six ab-initio reconstructions. To perform further discarding of bad particles, the heterogeneous refinement was processed by using the two dominant classes with the associated particles of the six initial volumes. Among the four 3D classes, one volume containing ~31.02% of total particles were subjected to the homogeneous refinement and iterative global CTF refinement was performed, yielded a final density map at 3.05 Å resolution estimated by the gold-standard Fourier shell correlation (FSC) 0.143 criterion. The final map was sharpened by DeepEMhancer.

The prototype TGEV RBD-dAPN complex dataset was processed similarly. In another initially collected dataset, we picked out particles using blob-pick procedure of cryoSPARC from 1,000 micrographs, and then these particles were subjected to 2D classification. After three rounds of 2D classification, we selected good particles in different views for Topaz training and then generated the Topaz model. Then we applied the Topaz procedure to select particles against entire micrographs, The 1,933,388 initial particles were picked and extracted from 21,536 micrographs. After the extensive 2D classification, approximately 3,600,584 good particles were selected to generate the initial models and 3D classification and resulted to four distinct volumes. The two dominant class containing 30.6% and 10.89% of total particles was identified, which displayed clear features of secondary structural elements, especially in the area of the binding interface of the TEGV RBD and dAPN. These particles were subjected to homogeneous, non-uniform and CTF refinements in cryoSPARC v.4.5.1, which yielded a final density map at 2.90 Å resolution estimated by the gold-standard Fourier shell correlation cut-off value of 0.143. We also conducted one round of local refinement focused on the RBD-APN binding interface to improve the map quality and finally yielded a 2.86 Å cryo-EM map for the prototype RBD-APN subcomplex structure. The final map was sharpened by DeepEMhancer.

### Model building and structure refinement

For the initial model building of the PDCoV RBD-dAPN complex, the PDCoV RBD-hAPN (PDB code 7VPQ) was used as the starting model and fitted into the corresponding cryo-EM maps using UCSF Chimera v.1.15 [47]. Mutation and manual adjustment were carried out with COOT v.0.9.3 [48]. Glycans were added at N-linked glycosylation sites in Coot. For the TEGV RBD-dAPN complex, model was built using published coordinates CCoV-HuPn-2018 RBD-dAPN ectodomain (PDB code 7U0L) with Phenix and Coot based on the two focused-refined cryo-EM maps, most residue side chains of which were clearly visible in the map. Each residue was manually checked with the chemical properties taken into consideration during model building. Several rounds of the real-space refinement in Phenix-1.20.1 [49] and manually building in Coot were performed until the final reliable models were obtained. Molprobity [50] was used to validate geometry and check structure quality. Statistics associated with data collection, 3D reconstruction and model building were summarized in **S1 Table**. Figures were generated using Chimera [47] and PyMol v.2.0 (http://www.pymol.org).

## Supporting information

S1 Table Cryo-EM data collection, refinement and validation statistics. (DOCX)

S2 Table The interaction of PDCoV RBD with dog APN. (DOCX)

S3 Table The interaction of TGEV RBD with dog APN. (DOCX)

S4 Table The accession numbers of the APN from 17 species. (DOCX)

S1 Fig Binding affinity assay between TGEV RBD and APN-797 mutants from bovine and sheep by SPR. The hFc-tagged APN mutants were captured by protein A chip, and sequentially tested the binding with serially diluted TGEV RBD. Mean ± SD represents the mean and standard deviation of three independent experiments. Actual and fitted curves are colored in black and red, respectively.
(TIF)

S2 Fig EM data processing of dog APN and PDCoV RBD complex. (A) Representative cryo-EM micrograph. (B) 2D class average images of dog APN and PDCoV RBD complex. (C) Fourier Shell Correlation (FSC) of final EM map and model vs. map. (D) Angular distribution of the particles for 3D reconstruction. (E) Flow chart of data processing and reconstruction.
(TIF)

S3 Fig EM data processing of dog APN and TGEV RBD complex. (A) Representative cryo-EM micrograph. (B) 2D class average images of dog APN and TGEV RBD complex. (C) Fourier Shell Correlation (FSC) of final EM map and model vs. map. (D) Angular distribution of the particles for 3D reconstruction. (E) Flow chart of data processing and reconstruction.
(TIF)

S4 Fig The complex structures of PDCoV RBD bound to pAPN, hAPN and dAPN. (A) The complex structure of PDCoV RBD bound to dAPN. PDCoV RBD and dAPN are colored in magenta and green, respectively. (B) The complex structure of PDCoV RBD bound to hAPN. PDCoV RBD and hAPN are colored in magenta and yellow, respectively. (C) The complex structure of PDCoV RBD bound to pAPN. PDCoV RBD and pAPN are colored in magenta and cyan, respectively. (D) Superposition of PDCoV RBD–dAPN (cyan) and PDCoV RBD–hAPN (magenta) complexes. The root mean square deviation (RMSD) between them is 1.22 Å. (E) Superposition of PDCoV RBD–dAPN (cyan) and PDCoV RBD–pAPN (orange) complexes. The root mean square deviation (RMSD) between them is 1.11 Å. (F) Superposition of TGEV RBD-dAPN (blue) and PRCoV RBD–pAPN (orange) complexes. The root mean square deviation (RMSD) between them is 1.50 Å.
(TIF)

S5 Fig Mutational analysis of S464C+S929C in pAPN involved in interaction with TGEV or PDCoV. (A) The hFc-tagged APN mutants were captured by protein A chip, and sequentially tested the binding with serially diluted TGEV RBD. Mean ± SD represents the mean and standard deviation of three independent experiments. Actual and fitted curves are colored in black and red, respectively. (B) TGEV infection in BHK-21 cells overexpressing the pAPN or its mutant S464C+S929C. Green fluorescence indicates BHK-21-APN cells infected with TGEV. The scale bar indicates 50 μm. (C) PDCoV infection in BHK-21 cells overexpressing the pAPN or its mutant S464C+S929C. Green fluorescence indicates BHK-21-APN cells infected with PDCoV. The scale bar indicates 50 μm. (D) The fluorescence intensity in (B) and (C) was determined by software Image J. Data are expressed as mean ± SD, n = 5. Error bars denote standard deviations for the samples.
(TIF)
