# Supplementary material for: Cross-species recognition of two porcine coronaviruses to their cellular receptor aminopeptidase N of dogs and seven other species
Source: PLoS Pathog. 2025 Jan 7;21(1):e1012836. doi: 10.1371/journal.ppat.1012836 (PMC11741606; doi:10.1371/journal.ppat.1012836)
Supplement: S1 Table — (DOCX) [file ppat.1012836.s001.docx]

**Table S1. Cryo-EM data collection, refinement and validation statistics**

|  | PDCoV RBD-Dog APN  (PDB 8YZI) | TEGV RBD-Dog APN  (PDB 8Z27) |
| --- | --- | --- |
| **Data collection and processing** |  |  |
| Magnification | 105k | 105k |
| Voltage (kV) | 300 | 300 |
| Electron exposure (e–/Å^2^) | 60 | 60 |
| Defocus range (μm) | -1.0 ~ -2.0 | -1.0 ~ -2.0 |
| Pixel size (Å) | 0.69 | 0.69 |
| Symmetry imposed | C1 | C1 |
| Initial particle images (no.) | 4,127,192 | 3,600,584 |
| Final particle images (no.) | 90,772 | 295,620 |
| Map resolution (Å)  FSC threshold | 3.05  0.143 | 2.86  0.143 |
|  |  |  |
| **Refinement** |  |  |
| Initial model used (PDB code) | 7VPQ | 7U0L |
| Model resolution range (Å) | Up to 3.05 | Up to 2.86 |
| Map sharpening *B* factor (Å^2^) | DeepEMhancer (no B factor value) | DeepEMhancer (no B factor value) |
| Model composition  Non-hydrogen atoms  Protein residues  Ligands | 16699  2030  ZN:2  BMA:1  NAG:26 | 17177  2098  BMA:2  NAG:20  FUC:2 |
| *B* factors (Å^2^)  Protein  Ligand | 45.13  50.83 | 0.32  50.00 |
| R.m.s. deviations  Bond lengths (Å)  Bond angles (°) | 0.004  0.953 | 0.008  1.664 |
| Validation  MolProbity score  Clashscore  Poor rotamers (%) | 1.42  4.41  0.17 | 2.99  57.76  7.73 |
| Ramachandran plot  Favored (%)  Allowed (%)  Disallowed (%) | 96.74  3.26  0.00 | 97.61  2.01  0.38 |
