# Supplementary material for: Cross-species recognition of two porcine coronaviruses to their cellular receptor aminopeptidase N of dogs and seven other species
Source: PLoS Pathog. 2025 Jan 7;21(1):e1012836. doi: 10.1371/journal.ppat.1012836 (PMC11741606; doi:10.1371/journal.ppat.1012836)
Supplement: S2 Table — (DOCX) [file ppat.1012836.s002.docx]

**Table S2.** **The interaction of PDCoV RBD with dog APN.**

| **dog APN** | **PDCoV RBD** |
| --- | --- |
| N321 | F318(2) |
| Y322 | F318(10) |
| R325 | D317(2), F318(3) |
| Y375 | F318(3, **1**) |
| Q378 | Y394(2), V395(2) |
| I382 | E320(1) |
| K385 | D317(1, **1**), F318(4, **1**), E320(4) |
| E432 | R322(1) |
| T434 | R322(5, **1**) |
| E435 | E320(5, **1**) |
| T749 | N397(4, **1**) |
| D750 | I391(1), N397(2), Y398(4), L399(10, **1**) |
| H751 | N397(2), Y398(2), L399(3, **1**) |
| Q753 | A321(1, **1**), L399(5), L400(3), R401(6, **1**) |
| T754 | R401(2) |
| P795 | W396(5) |
| I796 | W396(4) |
| Y797 | W396(7) |
| P798 | W396(1) |
| **Total** | **107, 10** |

Numbers in the parentheses beside the PDCoV RBD residues represent the number of vdw contacts between the indicated residue with dAPN. Numbers in parentheses beside either ligand residues represent the number of vdw contacts the indicated residues conferred. The numbers with underline suggest numbers of potential H-bonds between the pairs of residues. vdw contact was analyzed at a cutoff of 4.0 Å and H-bonds at a cutoff of 3.3 Å.
