# Supplementary material for: Cross-species recognition of two porcine coronaviruses to their cellular receptor aminopeptidase N of dogs and seven other species
Source: PLoS Pathog. 2025 Jan 7;21(1):e1012836. doi: 10.1371/journal.ppat.1012836 (PMC11741606; doi:10.1371/journal.ppat.1012836)
Supplement: S4 Table — (DOCX) [file ppat.1012836.s004.docx]

**Table S4. The accession numbers of the APN from 17 species**

| APN | Accession number |
| --- | --- |
| Dog APN | P79143 |
| Pig APN | P15145 |
| Cat APN | P79171 |
| Red fox APN | A0A3Q7SK15 |
| Giant panda APN | A0A7N5JPH8 |
| Bovine APN | P79098 |
| Chicken APN | O57579 |
| Human APN | P15144 |
| Rat APN | P15684 |
| Mouse APN | P97449 |
| Horse APN | XP_008508865.1 |
| Arabian camel APN | XP_031296423.1 |
| Goat APN | A0A452E2A2 |
| Sheep APN | W5PQH2 |
| Malayan pangolin APN | UPI0008130BB4 |
| Eurasian tree sparrow APN | XP_039579479.1 |
| Rhesus macaque APN | XP_001093727.3 |
