# Supplementary figures and images for: Cross-species recognition of two porcine coronaviruses to their cellular receptor aminopeptidase N of dogs and seven other species
Source: PLoS Pathog. 2025 Jan 7;21(1):e1012836. doi: 10.1371/journal.ppat.1012836 (PMC11741606; doi:10.1371/journal.ppat.1012836)

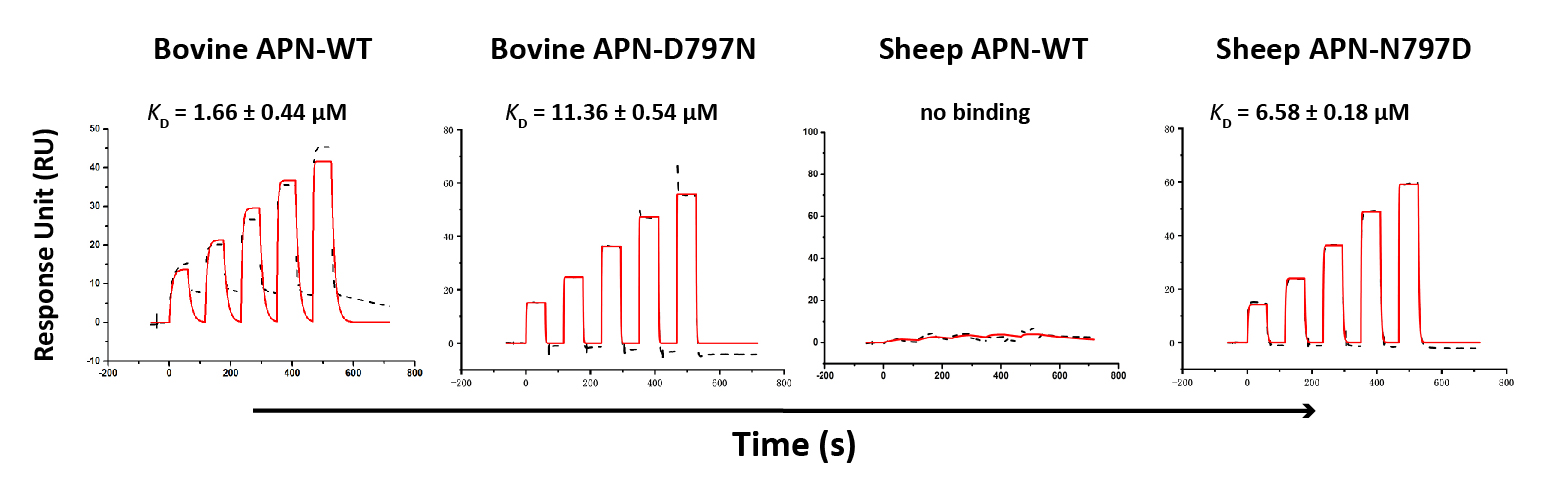

Supplement: S1 Fig — The hFc-tagged APN mutants were captured by protein A chip, and sequentially tested the binding with serially diluted TGEV RBD. Mean ± SD represents the mean and standard deviation of three independent experiments. Actual and fitted curves are colored in black and red, respectively. (TIF) [file ppat.1012836.s005.tif]

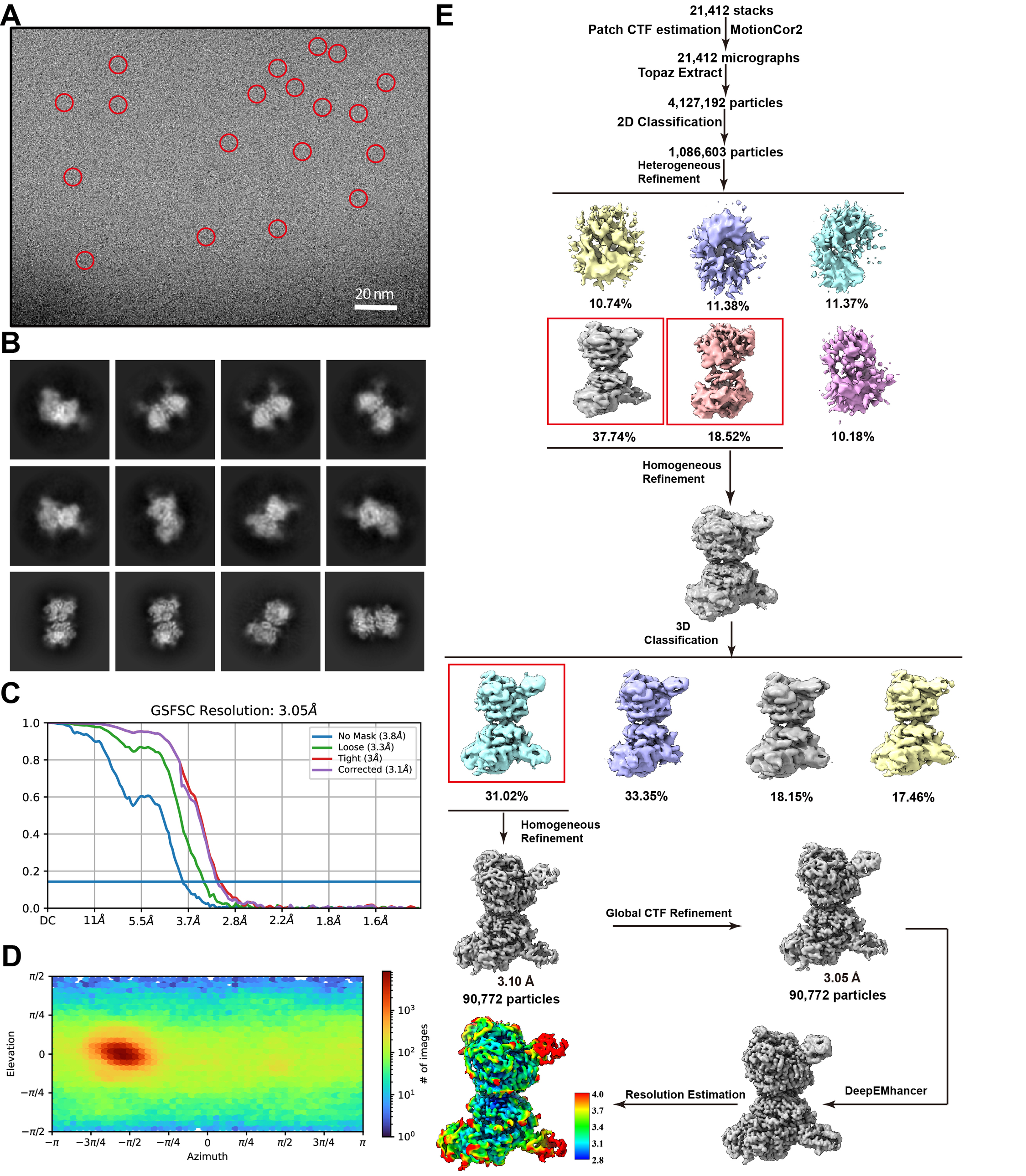

Supplement: S2 Fig — (A) Representative cryo-EM micrograph. (B) 2D class average images of dog APN and PDCoV RBD complex. (C) Fourier Shell Correlation (FSC) of final EM map and model vs. map. (D) Angular distribution of the particles for 3D reconstruction. (E) Flow chart of data processing and reconstruction. (TIF) [file ppat.1012836.s006.tif]

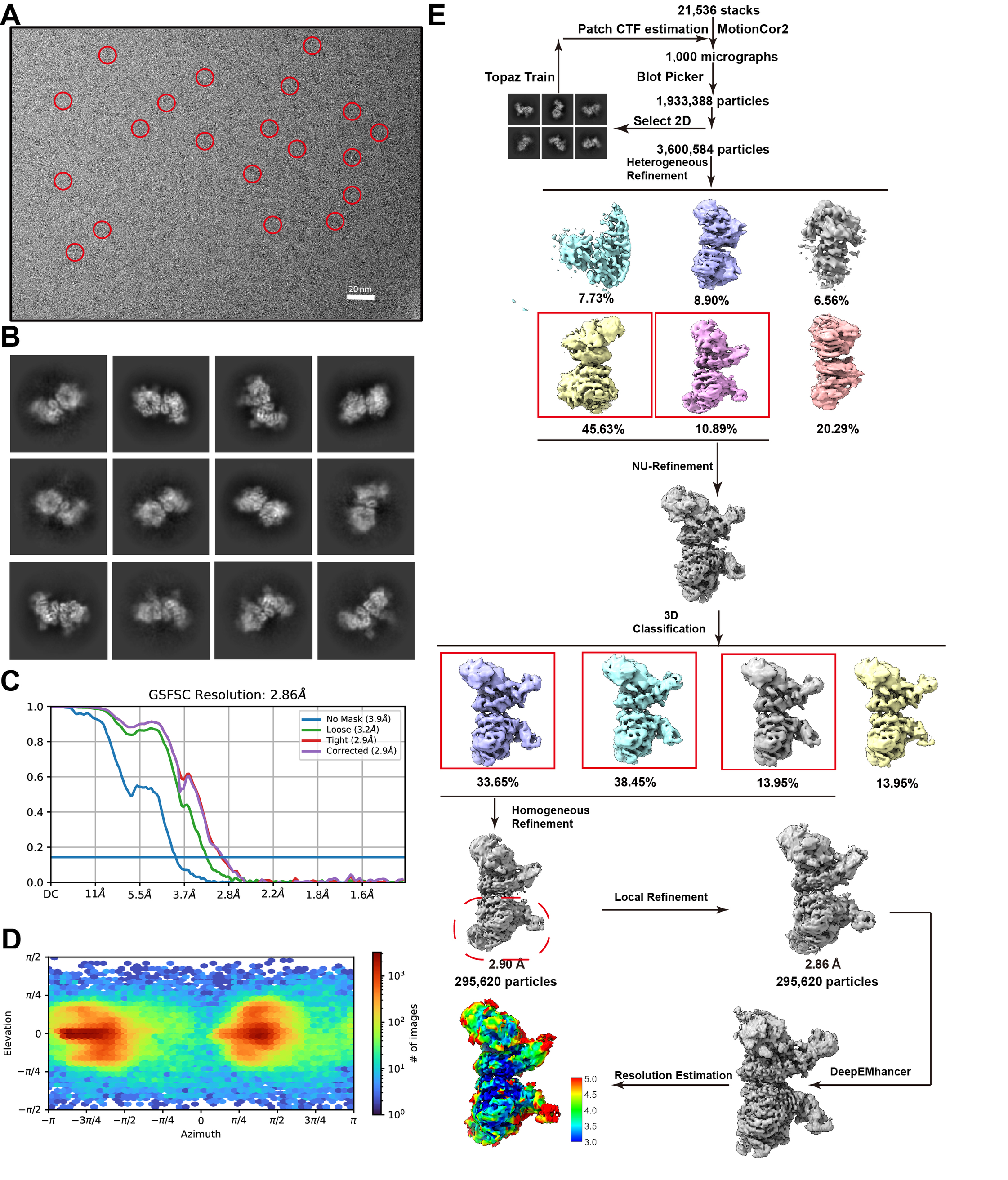

Supplement: S3 Fig — (A) Representative cryo-EM micrograph. (B) 2D class average images of dog APN and TGEV RBD complex. (C) Fourier Shell Correlation (FSC) of final EM map and model vs. map. (D) Angular distribution of the particles for 3D reconstruction. (E) Flow chart of data processing and reconstruction. (TIF) [file ppat.1012836.s007.tif]

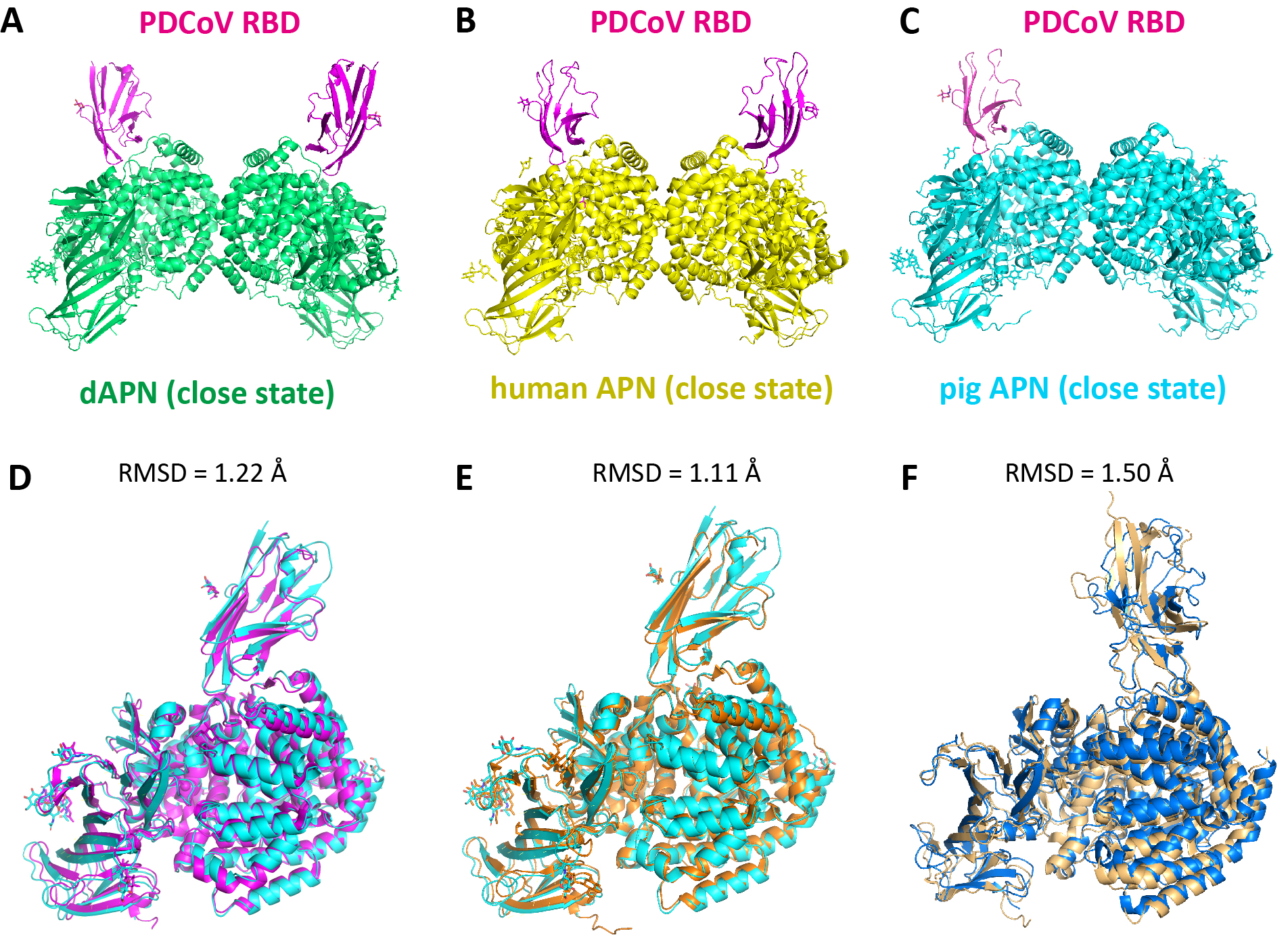

Supplement: S4 Fig — (A) The complex structure of PDCoV RBD bound to dAPN. PDCoV RBD and dAPN are colored in magenta and green, respectively. (B) The complex structure of PDCoV RBD bound to hAPN. PDCoV RBD and hAPN are colored in magenta and yellow, respectively. (C) The complex structure of PDCoV RBD bound to pAPN. PDCoV RBD and pAPN are colored in magenta and cyan, respectively. (D) Superposition of PDCoV RBD–dAPN (cyan) and PDCoV RBD–hAPN (magenta) complexes. The root mean square deviation (RMSD) between them is 1.22 Å. (E) Superposition of PDCoV RBD–dAPN (cyan) and PDCoV RBD–pAPN (orange) complexes. The root mean square deviation (RMSD) between them is 1.11 Å. (F) Superposition of TGEV RBD-dAPN (blue) and PRCoV RBD–pAPN (orange) complexes. The root mean square deviation (RMSD) between them is 1.50 Å. (TIF) [file ppat.1012836.s008.tif]

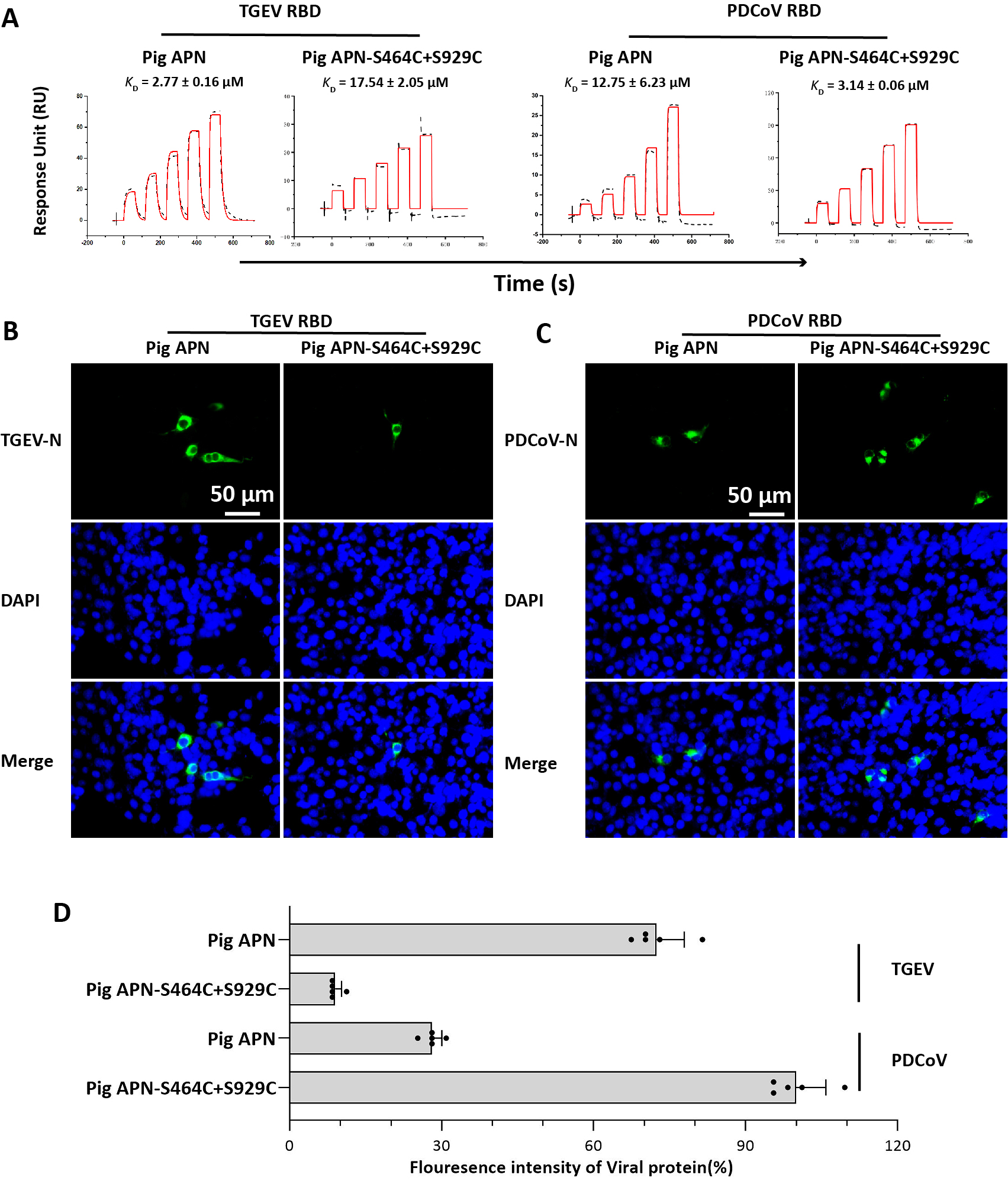

Supplement: S5 Fig — (A) The hFc-tagged APN mutants were captured by protein A chip, and sequentially tested the binding with serially diluted TGEV RBD. Mean ± SD represents the mean and standard deviation of three independent experiments. Actual and fitted curves are colored in black and red, respectively. (B) TGEV infection in BHK-21 cells overexpressing the pAPN or its mutant S464C+S929C. Green fluorescence indicates BHK-21-APN cells infected with TGEV. The scale bar indicates 50 μm. (C) PDCoV infection in BHK-21 cells overexpressing the pAPN or its mutant S464C+S929C. Green fluorescence indicates BHK-21-APN cells infected with PDCoV. The scale bar indicates 50 μm. (D) The fluorescence intensity in (B) and (C) was determined by software Image J. Data are expressed as mean ± SD, n = 5. Error bars denote standard deviations for the samples. (TIF) [file ppat.1012836.s009.tif]
